# Attitudes and personal beliefs about the COVID-19 vaccine among people with COVID-19: a mixed-methods analysis

**DOI:** 10.1186/s12889-022-14335-x

**Published:** 2022-10-18

**Authors:** Monica M. Bennett, Megan Douglas, Briget da Graca, Katherine Sanchez, Mark B. Powers, Ann Marie Warren

**Affiliations:** 1 Baylor Scott & White Research Institute, Dallas, TX USA; 2 Baylor University Medical Center, Dallas, TX USA; 3 School of Social Work, University of Texas at Arlington, Arlington, TX USA; 4 Texas A&M University – College of Medicine, Dallas, TX USA

**Keywords:** COVID-19, Vaccine, Religion, Greater good, Individual choice

## Abstract

**Background:**

Little research is available regarding vaccination attitudes among those recently diagnosed with COVID-19. This is important to investigate, particularly among those experiencing mild-to-moderate illness, given the ongoing need to improve uptake of both initial vaccine series and booster doses, and the divergent ways such an experience could impact attitudes.

**Methods:**

From September 3 – November 12, 2021, all patients enrolled in Baylor Scott & White’s “COVID-19 Digital Care Journey for Home Monitoring” were invited to participate in an online survey that included questions about vaccination status and attitudes/opinions regarding COVID-19 and the COVID-19 vaccines. Following an item asking about accordance of COVID-19 vaccination with religious/personal beliefs, participants were asked to describe those beliefs and how they relate to taking/not taking the vaccine.

**Results:**

Of 8,075 patients age ≥ 18 years diagnosed with COVID-19 and invited to join the survey during the study period, 3242 (40.2%) were fully vaccinated. In contrast, among the 149 who completed the questionnaire, 95(63.8%) reported full vaccination. Responses differed significantly between vaccination groups. The vaccinated group strongly agreed that COVID-19 is a major public health problem, the vaccines are safe and effective, and their decision to vaccinate included considering community benefit. The unvaccinated group responded neutrally to most questions addressing safety and public health aspects of the vaccine, while strongly disagreeing with statements regarding vaccine effectiveness and other preventative public health measures. The vaccinated group strongly agreed that taking the vaccine accorded with their religious/personal beliefs, while the unvaccinated group was neutral. In qualitative analysis of the free text responses “risk perception/calculation” and “no impact” of religious/personal beliefs on vaccination decisions were frequent themes/subthemes in both groups, but beliefs related to the “greater good” were a strong driver among the vaccinated, while statements emphasizing “individual choice” were a third frequent theme for the unvaccinated.

**Conclusion:**

Our results show that two of the three factors that drive vaccine hesitancy (complacency, and lack of confidence in the vaccines) are present among unvaccinated adults recently diagnosed with COVID-19. They also show that beliefs emphasizing the importance of the greater good promote public health participation.

**Supplementary information:**

The online version contains supplementary material available at 10.1186/s12889-022-14335-x.

## Introduction

Over the course of the COVID-19 pandemic, attitudes towards vaccination in the United States have shifted substantially. In early surveys, conducted before any COVID-19 vaccines were available, 65–78% of respondents reported being at least *somewhat likely* to accept a COVID-19 vaccine when it became available to them [1–6]. However, much lower vaccine acceptance rates were reported among racial/ethnic minorities, Republican voters, rural residents, members of lower income households, persons lacking health insurance, and individuals with less education [1–3, 5–9]. Over time, disparities in vaccine acceptance rates among races and ethnicities decreased [10] while disparities related to political leanings and education persisted [11, 12]. As of August 2022, only 77.1% of U.S. adults have completed the primary vaccine series [13], with 19% still indicating they will definitely not be vaccinated [14].

Although the news media are replete with stories of individuals who, after contracting COVID-19 and experiencing serious illness or loss of a loved one, express regret at not having been vaccinated [15–18], there is little research evidence regarding vaccine attitudes among people who have contracted COVID-19. Data from the Household Pulse Survey indicate that vaccination receipt was lower and reluctance to be vaccinated was higher among people with a past diagnosis of, or unsure if they previously had, COVID-19 [19, 20]. These data did not, however, allow for insight into reasons why that was the case – for example, differentiating between people who feel that having immunity from a previous infection will protect them versus people who experienced a mild case of COVID-19 and, based on that experience, are not concerned about either catching it again or infecting others. It is also not currently known how people who contract COVID-19 after being vaccinated feel about the vaccines – some may, for example, come to doubt the vaccines’ effectiveness based on their own experience, while for others the experience might emphasize the importance of vaccination for avoiding serious illness. Investigating vaccine attitudes among people who have had breakthrough infections can offer important insights for addressing the lag in booster uptake, currently sitting at 48% of those eligible for the first booster [13].

This manuscript presents survey responses regarding vaccination attitudes from adults surveyed ~ 7 days after receiving a COVID-19 diagnosis. Quantitative results cover Likert scale survey responses about attitudes/beliefs in 6 topic areas: COVID-19 risk, Resources and access to vaccines, Safety of the Covid-19 vaccines, Public health aspects of COVID-19, Influences on vaccination decisions (including religious/personal beliefs), and Confidence in protective measures against COVID-19 (including vaccine effectiveness). Qualitative results provide additional insight regarding the influence of religious/personal beliefs on vaccination decisions.

## Methods

Study Design, Sample and Data Collection: This was a cross-sectional observational survey-based study. The study protocol was approved by the Institutional Review Board (IRB) at Baylor Scott &White Research Institute (#020–139) and informed consent was obtained from each participant prior to enrolment. No compensation was provided for participation in the study.

Data were obtained from respondents to an online questionnaire comprised of multiple measures; the total survey took about 30 minutes to complete. From September 3, 2021 to November 12, 2021, corresponding to the second half of the delta wave in Texas [21], all adult (≥ 18 years) ambulatory care patients who tested positive for SarsCoV-2 at a Baylor Scott & White Health (BSWH) facility or were diagnosed with a suspected case of COVID-19 symptoms by a provider at our facilities were invited to participate in the “COVID-19 Digital Care Journey for Home Monitoring”, administered through the MyBSWHealth™ software application and patient portal. The communications sent on Day 7 of the digital care journey (corresponding approximately with the seventh day after diagnosis) included an invitation to participate in this research study. This recruitment strategy has been described in detail elsewhere [22]. Consent and study data were collected and managed using the Research Electronic Data Capture (REDCap™) platform, hosted at Baylor Scott & White Research Institute. REDCap™ is a secure, web-based platform designed for research data capture [23, 24]. The end date for inclusion in this analysis was based on substantial slowing of new enrollees during the first 2 weeks of November 2021 as new cases in the BSWH service areas declined.

### Quantitative variables

Demographic variables. Sociodemographic variables collected included age, gender, race, ethnicity, income, education, employment, and marital status. Participants were also presented with a list of medical and mental health conditions and asked to indicate whether or not they had been diagnosed with that condition, and, if they had, whether it was a past or current diagnosis.

COVID-19 Experience: Questions about COVID-19 experience also included whether they had been hospitalized or put on a ventilator due to COVID-19. Participants were also asked whether they had been vaccinated against COVID-19, and, if yes, with which vaccine product and when. Participants who responded no were asked if they would be vaccinated in the future (response options included *yes*, *no*, and *maybe*).

Fear of COVID-19 Scale. Perceived fear of COVID-19 was assessed with a 7-item validated scale [25] asking participants to indicate how much they agree or disagree (1 = *strongly disagree* and 5 = *strongly agree*) with items assessing fears and worries about Coronavirus-19. The total score is calculated by summing item responses and ranges from 7 to 35 with higher scores suggesting greater fear of COVID-19. The internal consistency, calculated as Chronbach’s alpha, for our sample was 0.89.

COVID-19 Stigma. Stigma related to COVID-19 was assessed using the Stigma Scale for Chronic Illness (SSCI-8), an 8-item questionnaire [26] initially created to measure perceived stigmatization among people with chronic illnesses. The questionnaire was adapted for COVID-19 by instructing participants to answer items “in reference to your COVID-19 screening status”. Items asked about perceived stigma situations related to ones’ illness with responses indicating how often each item occurred on a 5-point qualitative scale (ranging from 0 = *never* to 4 = *always*). All items are summed for a total score ranging from 0 to 32, with higher scores suggesting greater stigmatization. Summed scores are then converted to standardized t-scores [27]. The internal consistency in our sample was 0.89.

Vaccination beliefs. Participants were asked to evaluate 34 statements, developed through multiple iterations by the study team and based on a combination of evidence available at the time in the literature from broader population surveys regarding factors influencing people’s vaccination decisions (e.g., concerns about safety, effectiveness, and side effects which were raised during the months preceding and immediately following vaccine availability [9]) and issues being raised locally or in the media related to vaccination decisions (e.g., employer vaccine mandates and religious exemptions [28–30], and that lack of FDA approval of the COVID-19 vaccines was a reported reason for not yet taking the vaccine [31] ), indicating their agreement on a 0 to 4 scale ranging from “strongly disagree” to “strongly agree” with a “neutral” option in the middle. Covered topics related to risks associated with COVID-19, their ability to access COVID-19 vaccines, the safety of the COVID-19 vaccines, public health aspects of COVID-19/vaccination against COVID-19, influences on the decision to take/not take the COVID-19 vaccines (including religious/personal beliefs), and confidence in the protection against contracting COVID-19 that vaccination and non-pharmaceutical interventions such as masking and social distancing achieves. Participants were also asked to rate how frequently they had worn a mask when going out in public in the last 2 weeks on a 0 to 3 scale ranging from “none of the time” to “all of the time”. Each item was assessed individually. The internal consistency of the 34 statements was 0.93.

### Qualitative variables

The survey included one open-ended question immediately following the quantitative item asking respondents to rate their agreement with the following: “Taking the COVID-19 vaccine is in accordance with my religious/personal beliefs (0- strongly disagree to 4- strongly agree; 5 -prefer not to answer)”. For all participants, including those who selected ‘prefer not to answer’, a free response item then asked, “Please describe those beliefs and how they relate to taking/not taking the vaccine”, thus providing the opportunity to explain the relationship between those beliefs and their decision in their own words.

### Analysis

#### Quantitative analysis

A participant who received at least one dose of any of the approved COVID-19 vaccines was included in the vaccinated group. Participant demographic and COVID-related characteristics were compared using t-tests for continuous variables, Mann-Whitney U tests for ordinal variables, and chi-square or Fisher’s test for categorical variables. The ranking of vaccine attitudes was compared between groups using Mann-Whitney U tests. All analysis was performed using SAS 9.4.

#### Qualitative analysis

To analyze free text responses, a consensual qualitative research (CQR) approach[32] was used to derive meaning about the impact of personal or religious beliefs for participants based on whether they were vaccinated or not vaccinated. Coding was conducted by three study researchers (BdG, KS, MB; see reflexivity statements in the supplement for brief background) and overseen by a qualitative research trainer (MD). Additionally, an auditor was used for review and provided input when coders could not reach consensus (AMW). Data were divided into ‘vaccinated’ and ‘unvaccinated’ groups. All blank or non-text responses were removed prior to coding (n = 4). Coders and trainer met to form preliminary domains based on visual inspection of all responses (N = 145). Each response, or case, representing a single participant was inspected for fit into domains and could apply to multiple coding categories (e.g., 2, 3, or 4 codes). The coders individually reviewed each case looking for patterns and fit within initial domains. All 3 coders and the trainer then met to discuss rationale and reached consensus on all but 7 codes. Inter-rater agreement was calculated using Krippendorff’s alpha reliability coefficient [33] (alpha = 0.885), which exceeds the 0.823 cutpoint considered to indicate good agreement [34]. Responses were then sorted into each core idea and a final review was conducted to ensure fit within themes; consensus was reached on all domain and themes with the exception of 4 cases which were then reviewed by an auditor. The coders met one final time to discuss auditor’s input and were able to reach 100% consensus.

## Results

Between September 3, 2021 and November 12, 2021, 8,075 unique patients age ≥ 18 years tested positive for COVID-19 at a BSWH facility and thus received the invitation to participate in the COVID-19 Digital Health Journey through which participants for this research study were recruited [22]. Of those 8,075 patients, 4,122 (51.1%) were not vaccinated for COVID-19, 711 (8.8%) were partially vaccinated (i.e., had received 1 dose of a 2-dose regimen), and 3,242 (40.2%) were fully vaccinated.

There were 151 individuals who enrolled in the study between September 3, 2021 and November 12, 2021 and completed the vaccine questions. Two participants who indicated they had not been diagnosed with COVID-19 were removed. The proportion who had completed a primary vaccination series was substantially higher among study participants (69.1%) than in the eligible population of patients who received a COVID-19 diagnosis during this period (40.2%). Survey items with incomplete data are indicated in Table 1, and any missing data was excluded from analysis of that item.

### Quantitative results

Demographic characteristics and responses to COVID-19 questions stratified by vaccination status are summarized in Table 1.

**Table 1:** Characteristics of participants who enrolled in the study after receiving a COVID-19 diagnosis at a Baylor Scott & White Health facility between September 9, 2021 and November 12, 2021

	Total	Unvaccinated	Vaccinated	
	n = 149	n = 40	n = 109	**p-value**
**Age, mean** ± **sd**	52.0 ± 13.9	46.2 ± 12.9	54.1 ± 13.8	0.003
**Female sex**	118 (80.3%)	30 (78.9%)	88 (80.7%)	0.866
**BMI (n = 145), mean** ± **sd**	31.7 ± 8.3	32.2 ± 8.5	31.5 ± 8.2	0.779
*Categories*				0.984
Normal	31 (21.4%)	7 (18.9%)	24 (22.2%)	
Overweight	35 (24.1%)	9 (24.3%)	26 (24.1%)	
Obese	79 (54.5%)	21 (56.8%)	58 (53.7%)	
**Race**				0.328
White	119 (81%)	31 (81.6%)	88 (80.7%)	
Black	10 (6.8%)	1 (2.6%)	9 (8.3%)	
Hispanic	8 (5.4%)	4 (10.5%)	4 (3.7%)	
Other/unknown	10 (6.8%)	2 (5.3%)	8 (7.3%)	
**Married**	99 (67.3%)	23 (60.5%)	76 (69.7%)	0.221
**Education**				< 0.001
Highschool or less	17 (11.6%)	11 (28.9%)	6 (5.5%)	
Some college or Vocational/technical/Associates degree	52 (35.4%)	16 (42.1%)	36 (33%)	
Bachelor’s degree	43 (29.3%)	6 (15.8%)	37 (33.9%)	
Advanced degree	35 (23.8%)	5 (13.2%)	30 (27.5%)	
**Employed (n = 145)**	114 (76.5%)	29 (76.3%)	85 (78%)	0.742
**Income (n = 143)**				0.003
<$30,000	16 (11.2%)	7 (18.9%)	9 (8.5%)	
$30,000 - $60,000	29 (20.3%)	9 (24.3%)	20 (18.9%)	
$60,000 - $100,000	38 (26.6%)	13 (35.1%)	25 (23.6%)	
$100 - $150,000	35 (24.5%)	5 (13.5%)	30 (28.3%)	
>$150,000	25 (17.5%)	3 (8.1%)	22 (20.8%)	
**Current working status (n = 143)**				0.216
Working at my normal location	51 (35.2%)	11 (29.7%)	40 (37%)	
Working from home	32 (21.8%)	8 (21.6%)	24 (22.2%)	
Not working/Unemployed due to COVID-19	19 (13.1%)	8 (21.6%)	11 (10.2%)	
Not working right now for other reasons	43 (29.7%)	10 (27%)	33 (30.6%)	
**Any comorbidity** ^**1**^	96 (65.3%)	22 (57.9%)	74 (67.9%)	0.238
**Current or past smoker**	36 (24.5%)	8 (21.1%)	28 (25.7%)	0.690
**Any current mental health diagnosis**	59 (39.9%)	16 (42.1%)	43 (39.4%)	0.805
**Any past mental health diagnosis**	50 (27.5%)	12 (31.6%)	38 (34.9%)	0.661
**Covid experience**				
Hospitalized	11 (7.4%)	9 (22.5%)	2 (1.8%)	< 0.001
On ventilator	1 (0.7%)	1 (2.5%)	0 (0%)	NA
Stigma Scale T-score, mean ± sd	54.4 ± 8.6	57.3 ± 10.6	53.4 ± 7.5	0.042
COVID Fear, mean ± sd	16.5 ± 6.8	16.3 ± 7.6	16.6 ± 6.6	0.795
**Vaccine received**	-			
Pfizer	-	-	57 (52.3%)	-
Moderna	-	-	42 (38.5%)	-
Johnson & Johnson/Janssen	-	-	8 (7.3%)	-
Not specified	-	-	2 (1.8%)	-
**If two-dose vaccine, completed**	-	-	95 (96%)	-
**If one-dose vaccine, time since dose (days), median (IQR)**	-	-	191.5 (173.5, 197.5)	-
**If two dose vaccine, time since second dose (days), median (IQR)**	-	-	192 (163.5, 216.5)	-
**Time since fully vaccinated (time since last dose – 14 days), median (IQR)**	-	-	178 (149.5, 198.0)	-
**Intention to be vaccinated in the future**				
Yes	-	10 (25%)	-	-
Maybe	-	18 (45%)	-	-
No	-	12 (30%)	-	-

BMI = Body Mass Index; sd = standard deviation; IQR = interquartile range

^**1**^Comorbid conditions include chronic lung disease, diabetes mellitus, cardiovascular disease, chronic renal disease, liver disease, immunocompromised condition, neurologic/neurodevelopmental/ intellectual disability, traumatic brain injury, spinal cord injury, cancer, other chronic disease as identified by the participant

The vaccinated and unvaccinated groups differed significantly on age (p < 0.001; with the vaccinated group being older), education (p < 0.001; with the vaccinated group having completed higher levels of education), income (p = 0.003; with the vaccinated group earning higher), and COVID-19 experience (with the vaccinated group being less likely to have been hospitalized, p < 0.001; and to have experienced less stigma related to COVID-19, p = 0.042). Pfizer (52%) and Moderna (49%) were the most common vaccines taken with a median time since the last dose of 192 days. The majority of the unvaccinated group indicated they would or maybe would get the vaccine in the future (70%), while 30% maintained they will not get the COVID-19 vaccine.

Table 2 compares the vaccine beliefs between groups. All beliefs were statistically different between the two groups, with the exception of worry about COVID-19 variants.

**Table 2:** Vaccine attitudes rated on a scale of 0 – strongly disagree to 4 – strongly agree

	Unvaccinated		Vaccinated		
	n	median (Q1, Q3)	n	median (Q1, Q3)	**p-value**
**COVID-19 risk**					
Getting vaccinated is the best way to reduce risk for getting COVID-19	38	1.5 (0, 2)	109	4 (3, 4)	< 0.001
Getting vaccinated is the best way to reduce risk for getting severely ill with COVID-19	38	2 (1, 3)	108	4 (4, 4)	< 0.001
Before/without being vaccinated, my risk for catching COVID-19 was/is high	38	2 (1, 2)	109	4 (3, 4)	< 0.001
Before/without being vaccinated, my risk for becoming severely ill if I did catch COVID-19 was/is high	38	2 (0, 2)	109	4 (3, 4)	< 0.001
COVID-19 is a serious disease that can cause death or long-term symptoms even in healthy younger adults	38	3 (2, 4)	109	4 (4, 4)	< 0.001
How worried are you about the COVID-19 variants (0 = not at all worried to 4 = extremely worried)	39	2 (1, 3)	109	3 (1, 3)	0.073
**Resources and Access**					
I was/am able to take time off work/school or have someone else care for my family for a few days if I experience side effects from a COVID-19 vaccine	39	3 (2, 4)	107	3 (3, 4)	0.005
I knew/know where I can get a COVID-19 vaccine	40	3 (3, 4)	109	4 (4, 4)	< 0.001
I was/will be able to get a vaccination appointment at a convenient time and location	37	3 (2, 3)	109	4 (3, 4)	< 0.001
I knew/know how to get the vaccine without having to pay out-of-pocket	37	3 (2, 4)	109	4 (4, 4)	< 0.001
**Safety**					
The COVID-19 vaccines authorized/approved by the FDA are safe and effective for general use	37	2 (1, 2)	109	4 (2, 4)	< 0.001
There is not enough evidence that the COVID-19 vaccines are safe and effective for people like me	39	3 (1, 4)	109	1 (0, 2)	< 0.001
The COVID-19 vaccines having full FDA approval is important for my decision on being vaccinated	36	2.5 (1.5, 3)	109	1 (0, 2)	0.002
Women who are pregnant, breastfeeding, or trying to get pregnant should get the vaccine	37	2 (1, 2)	106	3 (2, 4)	< 0.001
Getting COVID-19 is worse that the side effects of the COVID-19 vaccines	37	2 (2, 3)	109	4 (3, 4)	< 0.001
The risks of severe illness or death from COVID-19 are greater than risks of harm from the COVID-19 vaccines	37	2 (2, 3)	109	4 (3, 4)	< 0.001
**Public Health**					
COVID-19 vaccines are our best chance for getting back to normal	38	2 (0, 2)	109	4 (3, 4)	< 0.001
COVID-19 is a major public health problem	38	3 (2, 4)	109	4 (4, 4)	< 0.001
People who had COVID-19 still need to get the vaccine	37	2 (1, 3)	109	4 (2.5, 4)	< 0.001
When deciding whether to take a vaccine, I consider both my individual risk and benefits and those of my community	37	2 (2, 3)	109	4 (3, 4)	< 0.001
Protecting public health during a pandemic is more important than personal freedom	38	2 (1, 2)	109	4 (2, 4)	< 0.001
I have been vaccinated against other preventable diseases	39	3 (3, 4)	109	4 (4, 4)	< 0.001
How likely are you to get a COVID-19 vaccine booster shot if recommended by the CDC/FDA? (0 = not at all likely to 4 = extremely likely)	37	1 (0, 2)	109	4 (2, 4)	< 0.001
**Influences**					
My decision on being vaccinated would not be different if my employer/school required it	37	3 (2, 4)	108	4 (3, 4)	< 0.001
My decision on being vaccinated would not be different if my employer/school/state offered a bonus or other prize for it	37	3 (2, 4)	109	4 (3, 4)	0.004
My friends and family encouraged me to take the COVID-19 vaccine	39	2 (1, 3)	109	3 (2, 4)	< 0.001
My healthcare provider encourages vaccination against COVID-19 for everyone eligible for the vaccines	39	3 (2, 3)	108	4 (3, 4)	< 0.001
Taking the COVID-19 vaccine is an accordance with my religious/personal beliefs	35	2 (1, 2)	108	4 (2, 4)	< 0.001
**Confidence in Protective Measures**, **0 = not at all confident to 4 = extremely confident**					
How confident are you that getting a COVID-19 vaccine will prevent you from getting COVID?	39	0 (0, 2)	109	1 (0, 3)	0.011
How confident are you that wearing a mask will prevent you from getting COVID?	39	1 (0, 2)	109	2 (1, 3)	0.004
How confident are you that social distancing will prevent you from getting COVID?	39	2 (1, 3)	109	3 (1, 3)	0.011
How confident are you that getting a COVID-19 vaccine will prevent others from getting COVID?	38	1 (0, 2)	109	2 (1, 3)	< 0.001
How confident are you that wearing a mask will prevent others from getting COVID?	39	1 (0, 2)	109	2 (1, 3)	0.004
How confident are you that social distancing will prevent others from getting COVID?	39	2 (1, 3)	109	3 (1, 3)	< 0.001
**In the past two weeks, how often do you wear a mask around others/in public?**	40		109		0.009
None of the time		4 (10%)		2 (1.8%)	
Some of the Time		9 (22.5%)		15 (13.8%)	
Most of the time		10 (25%)		22 (20.2%)	
All of the time		17 (42.5%)		70 (64.2%)	

The vaccinated group more strongly agreed with statements about vaccines reducing risk of getting or suffering severe illness with COVID-19 (all p < 0.001). The vaccinated group also indicated greater knowledge of where to get a vaccine, better access to it, and greater agreement that the vaccines are safe (all p < 0.01).

When considering the evidence of the safety and effectiveness of the vaccine, the vaccinated group disagreed more than the unvaccinated group that evidence of the safety and effectiveness of the vaccine was lacking, and that FDA approval was important to their vaccination decision (all p < 0.01). The vaccinated group strongly agreed that COVID-19 was a major public health problem, and that their decision to vaccinate should also include the benefit to the community (all p < 0.001). In contrast, the median response for the unvaccinated group to most questions addressing safety and public health aspects of the vaccine, including considering community in their vaccination decision, was neutral. The unvaccinated group also indicated stronger disagreement regarding the effectiveness of vaccines in preventing COVID-19, likelihood that they will get recommended booster doses, and confidence in public health measures such as wearing masks or social distancing to prevent individual infection with, or community spread of COVID-19. Further, approximately 85% of the vaccinated group wore a mask most or all of the time around others or in public in the past two weeks compared to 68% of the unvaccinated group (p = 0.009).

Regarding factors influencing vaccination decisions, both groups agreed that their decision would not be different if a vaccine was required or incentivized by their employer, school, or state, with the vaccinated group having higher levels of agreement. The vaccinated group had more encouragement to get vaccinated from family and friends (p < 0.001) and health care providers (p < 0.001), and strongly agreed that taking the vaccine was in accordance with their religious/personal beliefs compared to over half (57%) of the unvaccinated providing a neutral response (p < 0.001).

### Qualitative results

Final coding themes, subthemes, descriptions, frequencies, and examples to the open-ended item inquiring about how religious/personal beliefs relate to taking or not taking the vaccine are presented in Table 3.

**Table 3:** Qualitative themes and subthemes for the Unvaccinated Sample (29 total responses; 47 codes applied) and Vaccinated Sample (99 total responses; 162 codes applied)

		Vaccinated			Unvaccinated	
**Theme**	**Subtheme and Description**	**n (%)**	**Examples**		**n (%)**	**Examples**
Religious/Personal Beliefs						
	References Religion: Mentions a specific religion (e.g. Christianity, Judaism), quotes scripture, or mentions religious beliefs generally	25 (15%)			9 (19%)	
			*I am Lutheran, so there were no prohibitions or encouragements. It is a personal decision*			*I am christian and it has no effect on my vaccination status*
			*Love of Neighbor; Clothe the poor and feed the hungry, support the widow*			*My body is my temple.*
	References God: References God specifically	7 (4%)			2 (4%)	
			*I believe God provides scientist with abilities to develop vaccines*			*mRNA will effect the human genome. I’m created in the image of God.*
			*I was not worried about taking the vaccine. My faith is in God.*			
	No Impact: References religious beliefs in the context of not impacting the decision to get vaccinated or not get vaccinate.	22 (13%)			7 (15%)	
			*My religion didn’t have anything to do with me getting the vaccine.*			*My being a born again Christian has nothing to do with getting vaccinated*
			*My faith is open to all medical procedures and treatments*			*The decision is not based on religion*
	Not Religious	3 (2%)			0 (0%)	
Community versus Self						
	Greater Good: References betterment of a group larger than the self (e.g. family, another person, community)	40 (25%)			0 (0%)	
			*I wanted it, to protect my family and to show them it is okay yo get vaccinated*			
			*Christian values call for loving your neighbor as yourself.*			
	Emphasizes Individual Choice: Emphasizes the importance of individual decision-making	8 (5%)			5 (11%)	
			*I believe it is a personal decision and it does not go against my religion or beliefs.*			*It is my choice as what I do with my body*
			*I think get the Covid vaccine is a personal choice you make. You should consult your doctor and decide for yourself. Don’t let the media or government dictate whether you get it or not.*			*It should be an individual’s decision to get it or not*
Medical						
	Risk Perception/ Calculation: Decision is based on perceived individual risk to COVID-19 and generally weighing that risk against other factors (e.g. vaccine effectiveness)	25 (15%)			8 (17%)	
			*I didn’t want to get sick.*			*Worried because of my underlying health conditions.*
			*COVID-19 is clearly a disease that will be reduced/eradicated only through herd immunity supported by vaccination. While there is a risk in the vaccine, for most people, this is less than the disease itself.*			*I do not think the vaccine is against my beliefs. I just don’t see that they work when vaccinated people are getting just as sick as unvaccinated people, in my opinion*
	Doctor’s Advice: References decision is impacted by doctor’s (or other healthcare workers) advice	4 (2%)			0 (0%)	
			*All should take unless advised not to by doctor or the religion*			
			*I understand science and value using it. I trust our health care professionals. I deeply care about other people.*			
	Need More Information/ Research: Expresses uncertainty about vaccine due to lack of adequate research or information.	1 (1%)			6 (13%)	
			*I do not trust it. To much information and nothing is concrete. Only got the vaccine for my job.*			*I don’t believe that they have tested it long enough to prove it works*
						*Not sure what to believe.*
	References Side Effects: Mentions specific side effects associated with getting the vaccine.	1 (1%)			2 (4%)	
			*I believe many vaccines work to keep people healthy but I am worried about side effects such as blood clots with the covid vaccine*			*I once got the flu shot and that year I ended up with the worst pneumonia I’ve ever had in my life right after. So this shot and it’s side effects worried me. I made it nearly the whole pandemic without getting sick so I felt safe*
						*previous blood clotting experience.*
Miscellaneous						
	Demonstrates Misinformation: Statement includes objectively false information about the vaccine	2 (1%)			3 (6%)	
			*this really isnt a vaccine, it is a special flu shot. if it wasnan vaccine, like smallpox, i wouldnt have gotten covid after receiving the shots*			*The so called vaccines are killing people*
			*I believe that Although the vaccination makes changes to our molecules it doesn’t significantly make changes that will harm our reproductive systems. For future generations.*			*mRNA will effect the human genome. I’m created in the image of God.*
	Belief in Science/ Vaccines: Decision is impacted by a belief or trust in scientific process or belief that vaccines are generally effective technology against disease (e.g. herd immunity)	15 (9%)			1 (2%)	
			*While I am a Christian, I believe the science and research that has gone into the development and testing of the vaccines. They are safe and effective.*			*It is my choice as what I do with my body. As with all medications you should only take fully approved medicines.*
			*I firmly believe that vaccines are a solid way to limit and potentially eradicate diseases. Anyone who disagrees is uneducated or misinformed and must be informed.*			
	References Mandate: References a vaccine mandate or being forced to get the vaccine from a larger system	4 (2%)			2 (4%)	
			*I do not trust it. To much information and nothing is concrete. Only got the vaccine for my job.*			*Being forced to take a vaccine is against the constitution.*
			*I think get the Covid vaccine is a personal choice you make. You should consult your doctor and decide for yourself. Don’t let the media or government dictate whether you get it or not*			*Our worldwide church has urged all members to get the vaccine and to wear mask. I do not believe it is a right of the church or government to enforce or mandate forms of medical care. It should be a personal choice made between a person and God.*
	Uncoded: Did not receive a code due to ambiguity or lack of content.	5 (3%)			2 (4%)	
			*I think people are gonna do what they wanna do.*			*im not taking*
			*I am vaccinated*			*None*

*Note*. Percentage is the calculated by dividing the count of that subtheme by the total number of codes (n = 47 unvaccinated; 162 vaccinated) since some responses (n = 14 unvaccinated; 53 vaccinated) received multiple codes. 29 unvaccinated and 99 vaccinated total responses includes all text responses (e.g. blanks or symbols removed; n = 2 unvaccinated and 2 vaccinated). Additionally, “NA” or “not applicable” responses were also excluded from coding (n = 9 unvaccinated; 10 vaccinated

Responses and counts (%) are grouped by vaccination status. Primary themes for both groups included: (1) Religious/Personal Beliefs, (2) Community versus Self, (3) Medical, and (4) Miscellaneous. A complete list of items under each theme is provided in Supplementary Tables 1 and 2 for the unvaccinated and vaccinated groups, respectively.

The unvaccinated sample included 29 total cases, and 47 codes were applied based on observed themes and subthemes. The most frequent subthemes in this group were ‘references religion’ (19%; e.g., *My body is my temple.*), ‘risk perception/ calculation’ (17%; e.g., *I do not think the vaccine is against my beliefs. I just don’t see that they work when vaccinated people are getting just as sick as unvaccinated people, in my opinion*), indicating that their religious or personal beliefs had ‘no impact’ on their vaccination decision (15%; e.g., *My being a born again Christian has nothing to do with getting vaccinated*), ‘needs more information/research’ (13%; e.g., *I don’t believe that they have tested it long enough to prove it works*), and ‘emphasizes individual choice’ (11%; e.g., *It is my choice as what I do with my body*). Many of the responses in the unvaccinated group, although mentioning religion, stated that their religion did not influence their decision and were double coded as both ‘religion having no impact’ or ‘referencing religion’ and ‘individual choice’ (e.g., *Our worldwide church has urged all members to get the vaccine and to wear mask. I do not believe it is a right of the church or government to enforce or mandate forms of medical care.*).

The vaccinated sample included 99 cases, and 163 codes were applied. The most frequent subthemes in this group were ‘greater good,’ meaning a reference to the betterment of a group larger than the self (such as family or community, e.g., *I wanted it, to protect my family and to show them it is okay [to] get vaccinated*), with 25% of responses indicating this influenced their choice. The next top three frequently cited themes were similar to the unvaccinated group, although endorsements were made in the opposite direction: ‘references religion’ (15%; e.g., *Love of Neighbor; Clothe the poor and feed the hungry, support the widow*), ‘risk perception/calculation’ (15%; e.g., *COVID-19 is clearly a disease that will be reduced/eradicated only through herd immunity supported by vaccination. While there is a risk in the vaccine, for most people, this is less than the disease itself*), or ‘no impact’ of religious or personal beliefs on the decision to get vaccinated (13%; e.g., *My faith is open to all medical procedures and treatments*). The next most frequently referenced subtheme was ‘belief in science/vaccines’ which suggests that their decision was impacted by a belief or trust in the scientific process (9%; e.g., *While I am a Christian, I believe the science and research that has gone into the development and testing of the vaccines. They are safe and effective*.). Similar to the unvaccinated group, many referenced their religious beliefs, but stated these did not have an impact on their decision (e.g., *My religion has nothing to do with this*). About half of the responses coded as ‘risk perception/calculation’ (12/25) also referenced the ‘greater good’ theme suggesting that this group also factored in other individuals’ perceived risk into their own risk calculation (e.g., *We should protect those that can’t protect themselves, I chose the vaccine so I didn’t infect 10 year old, baby granddaughters and other children/high risk people. I’m also high risk.*”). The ‘greater good’ theme was also dually referenced in some of the ‘religious’ themed responses (9/40; e.g., *As a Christian I try to help others. I try not to think of myself first*.) Frequencies of all overlapping codes are provided in Supplementary Tables 3 and 4 for the unvaccinated and vaccinated group, respectively.

## Discussion

In this mixed methods survey of adults who tested positive for COVID during the delta wave surge, we found distinctly divergent views on a range of beliefs and attitudes toward the COVID-19 vaccine. The vaccinated group strongly agreed that COVID-19 is a major public health problem, the vaccines are safe and effective, and their decision to vaccinate included considering community benefit. The unvaccinated group responded neutrally to most questions addressing safety and public health aspects of the vaccine, while strongly disagreeing with statements regarding vaccine effectiveness and other preventative public health measures. The vaccinated group strongly agreed that taking the vaccine accorded with their religious/personal beliefs, while the unvaccinated group was neutral. In qualitative analysis of the free text responses to the question asking participants to describe how their religious/personal beliefs relate to their decision to take/not take a COVID-19 vaccine, “risk perception/calculation” and “no impact” of religious/personal beliefs on vaccination decisions were frequent themes/subthemes in both groups, but beliefs related to the “greater good” were a strong driver among the vaccinated, while statements emphasizing “individual choice” were a third frequent theme for the unvaccinated.

The vaccinated group in our study sample was less likely to be hospitalized than the unvaccinated group, which is consistent with the evidence regarding the vaccines’ effectiveness against serious illness and related public health messaging [35] and may have contributed to the vaccinated participants’ beliefs about reduced risk of severe illness, greater agreement that the vaccines are safe, and that FDA approval was not necessary for them to take the vaccine. The recognition we found among the vaccinated that COVID-19 is a major public health problem and indication that their decision to vaccinate would benefit the community is similar to previous findings from the UK that one of the significant differences between vaccine-hesitant compared to vaccine-accepting individuals was a lower level of altruism in the former [36].

Our findings related to lower education and income in the unvaccinated patients with COVID-19 are consistent with known hesitations in these groups [12] and suggest a need for tailored messaging campaigns. Disagreement that vaccines are effective in preventing spread of COVID-19 and low confidence in public health measures such as wearing masks or social distancing was greatest among the unvaccinated group, despite their own COVID-19 infection, similar to findings in the UK and Ireland [37]. Though the majority of unvaccinated respondents did indicate they would be getting a vaccine in the future, the 30% who indicated they would not is substantially higher than the 19% of US adults overall who continue to report they definitely will not be vaccinated against COVID-19 [14]. As our sample predominantly captured individuals who experienced mild to moderate illness with COVID-19 able to be managed at home, it may be that the experience of mild illness reinforced perceptions that vaccination against COVID-19 is not necessary in this group. Also worth considering, however, is that opportunities may have been missed during these patients’ COVID-related healthcare encounters (including digital communications) for their providers to educate them about the safety and effectiveness of the COVID-19 vaccines available. Certainly, room for improvement in communication about vaccination is seen in the significantly lower agreement of the unvaccinated group towards the statement that “My healthcare provider encourages vaccination against COVID-19 for everyone eligible for the vaccines.” Further research would be needed to explore the mechanisms underlying this difference between vaccinated and unvaccinated groups. More specifically, future research could tease apart whether unvaccinated patients are seeing providers who are less likely to recommend vaccination for everyone eligible, whether providers are not discussing vaccinations with those they know to be opposed, or whether providers are giving the same information and recommendations to all their patients but the patients are perceiving it differently. Such work could provide valuable insight into effective strategies for addressing vaccine hesitancy, but unfortunately lies beyond the scope of the data collected for our study.

Numerous themes emerged from the qualitative responses which provide insight into how religious and personal beliefs relate to vaccination decisions. Importantly, in this sample of unvaccinated adults in the largest not-for-profit health system in Texas, we found mixed reporting of religious views influencing decisions, but with the vaccinated group more strongly agreeing that their religious/personal beliefs were in accordance with taking the COVID-19 vaccine. Further, many of those vaccinated mentioned their religious beliefs in conjunction with the concept of “greater good”, whereas the unvaccinated group was more likely to report that their religious beliefs had no impact in their vaccination decision – or, where it did, supporting the decision not to vaccinate as aligned with individual choice. Previous studies using US samples have reported that beliefs in an engaged God are associated with greater mistrust in the COVID-19 vaccine, an effect amplified among those with lower educational achievement [38]. Leaders and followers of various world religions, including Judaism, Protestant Christianity, and Catholicism, have been known to decline other vaccines due to the belief that it interferes with God’s will or faith in divine protection and healing [39, 40]. More specific religious objections have also been identified, such as objections among Muslims related to porcine or non-halal ingredients [41–43], as well as Ramadan fasting (and the risk that adverse vaccine reactions could lead to breaking the fast) [44], and among Catholics related to the use of cell lines derived from aborted fetuses in vaccine production [45, 46]. Similar religious taboos have been found among the reasons for non-vaccination among followers of Hinduism and Sikhism [43].

Other beliefs expressed by the unvaccinated respondents suggested themes and subthemes beyond the question’s focus on religious/personal beliefs. These reflected themes found in recent reviews of COVID-19 vaccine hesitancy, including beliefs that the vaccines are not safe or effective and concerns about their rapid development [47]. Also common among unvaccinated respondents in this sample were themes related to personal choice and freedom, issues that have generated protests and unrest around the globe related to work and travel requirements for vaccination. These themes echo similar objections raised in the past against requirements for vaccination against other diseases, such as those seen in reaction to the smallpox vaccine [48, 49].

The vaccinated participants in the current sample appeared highly motivated by personal sense of duty to protect others. Even as it related to a risk calculation, their concerns encompassed their loved ones and people at high risk for bad outcomes from COVID infection. These findings align with qualitative research on collective problem solving which found civic duty to be a strong predictor of compliance and makes people almost immune to other people’s attitudes in a crisis [50]. Similar to research on voting practices, people with a strong sense of civic duty may view compliance with public health recommendations as a moral obligation that they must abide by to be a good citizen [51].

### Limitations

As with all surveys, this study is not without limitations. The qualitative responses were captured via an online survey free text response item, which might have reduced social desirability bias, but did not allow for clarification or follow-up questioning as done with interview style qualitative research. Additionally, bias may have impacted the qualitative coding process. We attempted to reduce this by (1) having one member of the team (trainer) with experience in qualitative research oversee the coding process without actually coding any items; (2) by involving different perspectives from three different coders and providing a brief summary of coder’s personal background and beliefs in an effort to be transparent about potential biases; and (3) by providing all raw responses in supplementary tables as well as descriptions and examples of each classification.

This study was also limited by selection bias. Although recruitment through the COVID Digital Health Care Journey has many advantages related to time, cost, and reach, as with most digital recruitment strategies, it has a tendency to result in overrepresentation of white, educated, and female participants, limitations which we have detailed elsewhere [22]. While the ~ 2% completion rate we saw among eligible patients is not dissimilar from the 4% reported by previous disease-specific research studies using patient portal messaging for recruitment [52], specific points at which potential participants may have been lost include: not having/not registering for or not interacting with the patient portal or application (all being more likely among groups with less access to high speed internet and lower technological literacy); experiencing more severe symptoms, which might have required hospitalization prior to receiving the research invitation on Day 7 of the digital care journey or have left the potential participant feeling too ill to interact with the survey (both more likely in unvaccinated individuals); and experiencing survey fatigue before reaching the vaccine attitudes and demographics questions needed for this analysis.

The substantial difference observed in the proportion of study participants who were fully vaccinated (69.2%), compared to the proportion of those eligible to participate who were fully vaccinated (40.2%) indicates selection bias away from the unvaccinated. Furthermore, unvaccinated individuals who did participate may have been more likely to be “pro-research” than the unvaccinated individuals who did not participate. If so, our results regarding differences in attitudes to the COVID-19 vaccines are likely underestimates compared to the population.

Finally, all the survey responses analyzed were collected during the Delta wave in late 2021. It is possible that different attitudes towards vaccination would have been found in earlier or later waves – although in which direction attitudes may have varied is open to speculation. During the Omicron surge in the winter of 2021/2022, for example, the highly contagious nature of this variant might have made some people view vaccines more favorably, while the lesser severity of many of the cases might have made others more inclined to view the vaccines as unnecessary.

### Implications for practice/policy/further research

Even prior to the current pandemic, the World Health Organization identified vaccine hesitancy as one of the greatest threats to global health [53]. The reasons people choose not to vaccinate are complex, with identified barriers including complacency, inconvenience, and lack of confidence. Our results showed indications of greater complacency and lack of confidence in vaccines among unvaccinated individuals diagnosed with COVID-19. We also found marked differences in the way vaccinated and unvaccinated individuals viewed the relationship between their religious/personal beliefs and their vaccination decision, with the former emphasizing beliefs related to contributing to the “greater good” while the latter reported no impact or emphasized beliefs related to individual choice. These differences existed even between respondents who identified themselves as belonging to the same religion, providing valuable insight into how emphasis of different aspects or priorities in a religion can influence healthcare and public health decisions.

## Electronic supplementary material

Below is the link to the electronic supplementary material.

Supplementary Material 1

## Data Availability

The quantitative data analyzed during the current study are not publicly available due to the inclusion of private health information, but are available from the corresponding author on reasonable request. All qualitative responses are included in the supplementary information.
