# Supplementary material for: Attitudes and personal beliefs about the COVID-19 vaccine among people with COVID-19: a mixed-methods analysis
Source: BMC Public Health. 2022 Oct 18;22:1936. doi: 10.1186/s12889-022-14335-x (PMC9579584; doi:10.1186/s12889-022-14335-x)
Supplement: Supplementary file 1 — Supplementary Material 1 [file 12889_2022_14335_MOESM1_ESM.docx]

**Appendix**

Supplemental Table 1. Responses for unvaccinated participants grouped by theme and subtheme.

| **Theme** | **Subtheme** | **Response** |
| --- | --- | --- |
| Religious/ Personal Beliefs | |  |
|  | References Religion | 1. My being a born again Christian has nothing to do with getting vaccinated  2. My religious beliefs doesn't stop me from taking the vaccine  3. I am christian and it has no effect on my vaccination status.  4. My body is my temple.  5. My faith is none of your business.  6. I am Roman Catholic We have a choice of getting vaccinated  7. Our worldwide church has urged all members to get the vaccine and to wear mask. I do not believe it is a right of the church or government to enforce or mandate forms of medical care. It should be a personal choice made between a person and God.  8. I require more research within my religion before I can answer this  9. The decision is not based on religion |
|  | References God | 1. mRNA will effect the human genome. I'm created in the image of God.  2. Our worldwide church has urged all members to get the vaccine and to wear mask. I do not believe it is a right of the church or government to enforce or mandate forms of medical care. It should be a personal choice made between a person and God. |
|  | No Impact | 1. I dont have any spiritual beliefs about it  2. My being a born again Christian has nothing to do with getting vaccinated  3. My religious beliefs doesn't stop me from taking the vaccine  4. I am christian and it has no effect on my vaccination status.  5. I do not think the vaccine is against my beliefs. I just don't see that they work when vaccinated people are getting just as sick as unvaccinated people, in my opinion.  6. To be honest, I am just not comfortable with the vaccine yet. Nothing is making that decision for me. It's just how I feel to my own instinct. I understand medical professionals find it to be safe and that many people are perfectly fine. But I'm still not there in the comfort zone yet.  7. The decision is not based on religion |
|  | Not Religious | *NONE* |
| Community versus Self | |  |
|  | Greater Good | *NONE* |
|  | Emphasizes Individual Choice | 1. It is my choice as what I do with my body. As with all medications you should only take fully approved medicines.  2. I am Roman Catholic We have a choice of getting vaccinated  3. Our worldwide church has urged all members to get the vaccine and to wear mask. I do not believe it is a right of the church or government to enforce or mandate forms of medical care. It should be a personal choice made between a person and God.  4. The preservation of life is paramount. Individuals' beliefs and reasoned conclusions about how they can best preserve their own life and maintain proper concern for their fellow humans should be respected, even if you disagree.  5. It should be an individual's decision to get it or not |
| Medical | |  |
|  | Risk Perception/ Calculation | 1. I believe a person's own immune system was built to deal with an illness like this one.  2. previous blood clotting experience.  3. I do not think the vaccine is against my beliefs. I just don't see that they work when vaccinated people are getting just as sick as unvaccinated people, in my opinion.  4. I once got the flu shot and that year I ended up with the worst pneumonia I've ever had in my life right after. So this shot and it's side effects worried me. I made it nearly the whole pandemic without getting sick so I felt safe.  5. Worried because of my underlying health conditions.  6. I wasnt going to get the shot. I got severely ill with Covid. Im going to get vaccinated now unvaccinated group showed an intention to be vaccinated after recovery  7. The preservation of life is paramount. Individuals' beliefs and reasoned conclusions about how they can best preserve their own life and maintain proper concern for their fellow humans should be respected, even if you disagree.  8. To be honest, I am just not comfortable with the vaccine yet. Nothing is making that decision for me. It's just how I feel to my own instinct. I understand medical professionals find it to be safe and that many people are perfectly fine. But I'm still not there in the comfort zone yet. |
|  | Doctor’s Advice | *NONE* |
|  | Need More Information/ Research | 1. I don't believe that they have tested it long enough to prove it works  2. Too many uncertain issues (DNA/RNA) regarding the shot  3. To be honest, I am just not comfortable with the vaccine yet. Nothing is making that decision for me. It's just how I feel to my own instinct. I understand medical professionals find it to be safe and that many people are perfectly fine. But I'm still not there in the comfort zone yet.  4. Heard bad things and good things nor sure  5. I require more research within my religion before I can answer this  6. Not sure what to believe. |
|  | References Side Effects | 1. previous blood clotting experience.  2. I once got the flu shot and that year I ended up with the worst pneumonia I've ever had in my life right after. So this shot and it's side effects worried me. I made it nearly the whole pandemic without getting sick so I felt safe. |
| Miscellaneous | |  |
|  | Demonstrates Misinformation | 1. mRNA will effect the human genome. I'm created in the image of God.  2. The so called vaccines are killing people  3. I do not think the vaccine is against my beliefs. I just don't see that they work when vaccinated people are getting just as sick as unvaccinated people, in my opinion. |
|  | Belief in Science/ Vaccines | 1. It is my choice as what I do with my body. As with all medications you should only take fully approved medicines. |
|  | References Mandate | 1. Being forced to take a vaccine is against the constitution.  2. Our worldwide church has urged all members to get the vaccine and to wear mask. I do not believe it is a right of the church or government to enforce or mandate forms of medical care. It should be a personal choice made between a person and God. |
|  | Uncoded | 1. im not taking  2. None |
|  |  |  |

Supplemental Table 2. Responses for vaccinated participants grouped by theme and subtheme.

| **Theme** | **Subtheme** | **Response** |
| --- | --- | --- |
| Religious/ Personal Beliefs | |  |
|  | References Religion | 1. My religious beliefs have nothing to do with me getting the vaccine. It was a personal choice and should be a personal choice, not required by a government or business.  2. My religion didn't have anything to do with me getting the vaccine.  3. I am Lutheran, so there were no prohibitions or encouragements. It is a personal decision. (I  4. I believe I am responsible to serve and protect and consider others more important than myself according to Scripture. Therefore I should be concerned and care about others.  5. I don't think my religious beliefs play a part either way.  6. My faith/personal religion does not effect my views on the vaccine  7. As a Christian I try to help others. I try not to think of myself first.  8. I believe it is a personal decision and it does not go against my religion or beliefs.  9. Independent of my faith.  10. Love of Neighbor; Clothe the poor and feed the hungry, support the widow  11. My faith has little to do with the vaccine. Not liking this question.  12. Religious affiliation doesn't take a stand on this.  13. Health is paramount in the Jewish faith.  14. All should take unless advised not to by doctor or the religion  15. Christian  16. Christian values call for loving your neighbor as yourself.  17. I do not use my religious beliefs to inform my health decisions but my personal beliefs about preventative health care are my priority regarding covid-19 vaccines  18. I'm a Christian, and I believe we are taught to treat others the way we want to be treated. I want others to get vaccinated to help protect themselves and others from becoming sick. That's one of the main reasons why I got vaccinated as soon as I could.  19. Judaism prioritizes taking action to save lives above all else and getting vaccinated is a lifesaving measure.  20. My faith is open to all medical procedures and treatments  21. My religion does not have issues against vaccinations.  22. My religion encouraged me to get vaccinated. And I really want to make a difference in spreading this disease  23. My religion has nothing to do with this  24. My religion is Norse and it doesn't apply to the vaccine.  25. While I am a Christian, I believe the science and research that has gone into the development and testing of the vaccines. They are safe and effective. |
|  | References God | 1. I believe God provides scientist with abilities to develop vaccines  2. Because I believe God has blessed us with the wisdom and ability to take care of ourselves and each other in a responsible way.  3. Believe God sent us the vaccine  4. God has given us free will and common sense.  5. God is in control.  6. I believe my body is a gift from my maker and I am responsible to maintain health and wellness, to best serve my God.  7. I was not worried about taking the vaccine. My faith is in God. |
|  | No Impact | 1. I have no strong beliefs about vaccinations for myself personally  2. It had nothing to do with it.  3. My religious beliefs have nothing to do with me getting the vaccine. It was a personal choice and should be a personal choice, not required by a government or business.  4. My religion didn't have anything to do with me getting the vaccine.  5. I am Lutheran, so there were no prohibitions or encouragements. It is a personal decision. (I  6. I don't think my religious beliefs play a part either way.  7. My faith/personal religion does not effect my views on the vaccine  8. No beliefs related to taking vavvine  9. No impact  10. no impact, I am free to do what I need todo.  11. Not an issue  12. That would not make my decision  13. I believe it is a personal decision and it does not go against my religion or beliefs.  14. Independent of my faith.  15. My faith has little to do with the vaccine. Not liking this question.  16. Religious affiliation doesn't take a stand on this.  17. I do not use my religious beliefs to inform my health decisions but my personal beliefs about preventative health care are my priority regarding covid-19 vaccines  18. My faith is open to all medical procedures and treatments  19. My religion does not have issues against vaccinations.  20. My religion has nothing to do with this  21. My religion is Norse and it doesn't apply to the vaccine.  22. No conflict with personal beliefs |
|  | Not Religious | 1. I'm not religious  2. I am not religious.  3. Non religious |
| Community versus Self | |  |
|  | Greater Good | 1. I wanted it, to protect my family and to show them it is okay yo get vaccinated  2. I believe I am responsible to serve and protect and consider others more important than myself according to Scripture. Therefore I should be concerned and care about others.  3. As a Christian I try to help others. I try not to think of myself first.  4. Good for the many  5. It was protect a family member but I would have gotten any way  6. Love of Neighbor; Clothe the poor and feed the hungry, support the widow  7. We should protect those that can't protect themselves, I chose the vaccine so I didn't infect 10yr old, baby granddaughters and other children/high risk people. I'm also high risk  8. Health is paramount in the Jewish faith.  9. All should take unless advised not to by doctor or the religion  10. As a veteran my responsibility is to protect myself, my family and my community to the best of my ability which includes not spreading a virus that could harm them.  11. Because I believe God has blessed us with the wisdom and ability to take care of ourselves and each other in a responsible way.  12. Christian values call for loving your neighbor as yourself.  13. Community is more important than self.  14. COVID-19 is clearly a disease that will be reduced/eradicated only through herd immunity supported by vaccination. While there is a risk in the vaccine, for most people, this is less than the disease itself.  15. I believe everyone should do their part to help control this virus.  16. I believe in treating others with compassion and empathy. I believe we have more in common than not, and communities have to be willing to support one another, even strangers, to have an ethical and equitable society.  17. I believe it is important to protect others in addition to myself by receiving immunization.  18. I believe people have a duty to protect the most vulnerable in society.  19. I believe that everyone should do everything possible (vaccines, masks, limit activities...) to help limit the spread of the virus.  20. I believe that I have an obligation to protect others.  21. I believe that I should protect myself as well as my community as best as possible and I felt that the Covid vaccine was a way to keep me out of the hospital if I got it  22. I believe the greater good is more important than the individual  23. I believe we are responsible for doing the right thing for ourselves, families and neighbors. Being vaccinated is the best thing for all.  24. I believe we're to care about others, the greater good. Lay our lives down.  25. I understand science and value using it. I trust our health care professionals. I deeply care about other people.  26. I want to everything I can to protect my health and that of my family and community.  27. I'm a Christian, and I believe we are taught to treat others the way we want to be treated. I want others to get vaccinated to help protect themselves and others from becoming sick. That's one of the main reasons why I got vaccinated as soon as I could.  28. In best interest of me and of the public  29. It helps me. It helps others.  30. It seemed to be the only logical way to reduce the damage done by Covid19  31. Judaism prioritizes taking action to save lives above all else and getting vaccinated is a lifesaving measure.  32. My belief is simply that I want to protect my health and that if the people around me  33. My personal belief is to listen to Science and do what I need to do to be safe for my family and community.  34. My religion encouraged me to get vaccinated. And I really want to make a difference in spreading this disease  35. Need to care for others by not exposing them to this health risk  36. Personally believe in taking care of myself, my family, and the greater good.  37. Take care of others.  38. Vaccines get the nation back to normal  39. Value life  40. Everyone should be vaccinated. I hope they figure out a safe vaccine for young children as well. Having COVID is very scary. |
|  | Emphasizes Individual Choice | 1. It was mandated - no personal freedom  2. My religious beliefs have nothing to do with me getting the vaccine. It was a personal choice and should be a personal choice, not required by a government or business.  3. I am Lutheran, so there were no prohibitions or encouragements. It is a personal decision. (I  4. no impact, I am free to do what I need todo.  5. I believe it is a personal decision and it does not go against my religion or beliefs.  6. I believe that of you want to have the vaccine then you get it and if you do not want to have it then you do not need to.  7. I think get the Covid vaccine is a personal choice you make. You should consult your doctor and decide for yourself. Don't let the media or government dictate whether you get it or not.  8. God has given us free will and common sense. |
| Medical | |  |
|  | Risk Perception/ Calculation | 1. I believed at the time that it was an effective way to minimize the severity of the illness. Since both myself and my wife caught COVID even though we were vaccinated, I still believe the vaccination does not guarantee not catching the virus but does minimize the illness.  2. I didn't want to get sick.  3. I just wanted to be protected  4. I took the vaccine due to having covid.  5. We should protect those that can't protect themselves, I chose the vaccine so I didn't infect 10yr old, baby granddaughters and other children/high risk people. I'm also high risk  6. As a veteran my responsibility is to protect myself, my family and my community to the best of my ability which includes not spreading a virus that could harm them.  7. COVID-19 is clearly a disease that will be reduced/eradicated only through herd immunity supported by vaccination. While there is a risk in the vaccine, for most people, this is less than the disease itself.  8. I believe in doing everything in my power to take care of myself.  9. I believe it is important to protect others in addition to myself by receiving immunization.  10. I believe my body is a gift from my maker and I am responsible to maintain health and wellness, to best serve my God.  11. I believe that Although the vaccination makes changes to our molecules it doesn't significantly make changes that will harm our reproductive systems. For future generations.  12. I believe that I should protect myself as well as my community as best as possible and I felt that the Covid vaccine was a way to keep me out of the hospital if I got it  13. I do not use my religious beliefs to inform my health decisions but my personal beliefs about preventative health care are my priority regarding covid-19 vaccines  14. I want to everything I can to protect my health and that of my family and community.  15. I was not worried about taking the vaccine. My faith is in God.  16. In best interest of me and of the public  17. It helps me. It helps others.  18. It seemed to be the only logical way to reduce the damage done by Covid19  19. My belief is simply that I want to protect my health and that if the people around me  20. Personally believe in taking care of myself, my family, and the greater good.  21. Science provided a vaccine to protect me, it would be foolish to resist it.  22. Strong believer in vaccines to prevent serious illness  23. We are vaccinated against the flu each year how is Covid really any different.  24. Did not want to get the disease  25. Everyone should be vaccinated. I hope they figure out a safe vaccine for young children as well. Having COVID is very scary. |
|  | Doctor’s Advice | 1. I think get the Covid vaccine is a personal choice you make. You should consult your doctor and decide for yourself. Don't let the media or government dictate whether you get it or not.  2. All should take unless advised not to by doctor or the religion  3. Do the things doctors tell you to do  4. I understand science and value using it. I trust our health care professionals. I deeply care about other people. |
|  | Need More Information/ Research | 1. I do not trust it. To much information and nothing is concrete. Only got the vaccine for my job. |
|  | References Side Effects | 1. I believe many vaccines work to keep people healthy but I am worried about side effects such as blood clots with the covid vaccine |
| Miscellaneous | |  |
|  | Demonstrates Misinformation | 1. this really isnt a vaccine, it is a special flu shot. if it wasnan vaccine, like smallpox, i wouldnt have gotten covid after receiving the shots  2. I believe that Although the vaccination makes changes to our molecules it doesn't significantly make changes that will harm our reproductive systems. For future generations. |
|  | Belief in Science/ Vaccines | 1. I believed at the time that it was an effective way to minimize the severity of the illness. Since both myself and my wife caught COVID even though we were vaccinated, I still believe the vaccination does not guarantee not catching the virus but does minimize the illness.  2. I believe God provides scientist with abilities to develop vaccines  3. COVID-19 is clearly a disease that will be reduced/eradicated only through herd immunity supported by vaccination. While there is a risk in the vaccine, for most people, this is less than the disease itself.  4. God has given us free will and common sense.  5. I believe in herd immunity from being vaccinated  6. I believe that everyone should do everything possible (vaccines, masks, limit activities...) to help limit the spread of the virus.  7. I firmly believe that vaccines are a solid way to limit and potentially eradicate diseases. Anyone who disagrees is uneducated or misinformed and must be informed.  8. I understand science and value using it. I trust our health care professionals. I deeply care about other people.  9. It seemed to be the only logical way to reduce the damage done by Covid19  10. My personal belief is to listen to Science and do what I need to do to be safe for my family and community.  11. Science provided a vaccine to protect me, it would be foolish to resist it.  12. science!  13. Strong believer in vaccines to prevent serious illness  14. We are vaccinated against the flu each year how is Covid really any different.  15. While I am a Christian, I believe the science and research that has gone into the development and testing of the vaccines. They are safe and effective. |
|  | References Mandate | 1. It was mandated - no personal freedom  2. My religious beliefs have nothing to do with me getting the vaccine. It was a personal choice and should be a personal choice, not required by a government or business.  3. I do not trust it. To much information and nothing is concrete. Only got the vaccine for my job.  4. I think get the Covid vaccine is a personal choice you make. You should consult your doctor and decide for yourself. Don't let the media or government dictate whether you get it or not. |
|  | Uncoded | 1. I am vaccinated  2. I don't know  3. I think people are gonna do what they wanna do.  4. Get the goddamn vaxx, you morons.  5. Reasonable actions needed to be taken. |

Supplemental Table 3. Frequencies of Multiple Codes (unvaccinated sample)

|  | | | | | | | | | | | | | |
| --- | --- | --- | --- | --- | --- | --- | --- | --- | --- | --- | --- | --- | --- |
|  | 1 | 2 | 3 | 4 | 5 | 6 | 7 | 8 | 9 | 10 | 11 | 12 | 13 |
| 1. References Religion | - |  |  |  |  |  |  |  |  |  |  |  |  |
| 1. References God | 1 | - |  |  |  |  |  |  |  |  |  |  |  |
| 1. No Impact | 4 |  | - |  |  |  |  |  |  |  |  |  |  |
| 1. Not Religious |  |  |  | - |  |  |  |  |  |  |  |  |  |
| 1. Greater Good |  |  |  |  | - |  |  |  |  |  |  |  |  |
| 1. Emphasizes Individual Choice | 2 | 1 |  |  |  | - |  |  |  |  |  |  |  |
| 1. Risk Perception/ Calculation |  |  | 2 |  |  | 1 | - |  |  |  |  |  |  |
| 1. Doctor’s Advice |  |  |  |  |  |  |  | - |  |  |  |  |  |
| 1. Need More Information/ Research | 1 |  | 1 |  |  |  | 1 |  | - |  |  |  |  |
| 1. References Side Effects |  |  |  |  |  |  | 2 |  |  | - |  |  |  |
| 1. Demonstrates Misinformation |  | 1 | 1 |  |  |  | 1 |  |  |  | - |  |  |
| 1. Belief in Science/ Vaccines |  |  |  |  |  | 1 |  |  |  |  |  | - |  |
| 1. References Mandate | 1 | 1 |  |  |  | 1 |  |  |  |  |  |  | - |
| 1. Uncoded |  |  |  |  |  |  |  |  |  |  |  |  |  |

*Note*. 29 responses were coded for the unvaccinated group. Of these responses, 14 received more than one code, 3 received three codes, and 1 item received 4 codes.

Supplemental Table 4. Frequencies of Multiple Codes (vaccinated sample)

|  | | | | | | | | | | | | | |
| --- | --- | --- | --- | --- | --- | --- | --- | --- | --- | --- | --- | --- | --- |
|  | 1 | 2 | 3 | 4 | 5 | 6 | 7 | 8 | 9 | 10 | 11 | 12 | 13 |
| 1. References Religion | - |  |  |  |  |  |  |  |  |  |  |  |  |
| 1. References God |  | - |  |  |  |  |  |  |  |  |  |  |  |
| 1. No Impact | 14 |  | - |  |  |  |  |  |  |  |  |  |  |
| 1. Not Religious |  |  |  | - |  |  |  |  |  |  |  |  |  |
| 1. Greater Good | 9 | 1 |  |  | - |  |  |  |  |  |  |  |  |
| 1. Emphasizes Individual Choice | 3 | 1 | 4 |  |  | - |  |  |  |  |  |  |  |
| 1. Risk Perception/ Calculation | 1 | 2 | 1 |  | 12 |  | - |  |  |  |  |  |  |
| 1. Doctor’s Advice | 1 |  |  |  | 2 | 1 |  | - |  |  |  |  |  |
| 1. Need More Information/ Research |  |  |  |  |  |  |  |  | - |  |  |  |  |
| 1. References Side Effects |  |  |  |  |  |  |  |  |  | - |  |  |  |
| 1. Demonstrates Misinformation |  |  |  |  |  |  | 1 |  |  |  | - |  |  |
| 1. Belief in Science/ Vaccines | 1 | 2 |  |  |  | 1 | 6 | 1 |  |  |  | - |  |
| 1. References Mandate |  |  | 1 |  |  | 3 |  | 1 | 1 |  |  |  | - |
| 1. Uncoded |  |  |  |  |  |  |  |  |  |  |  |  |  |

*Note*. 99 responses were coded for the vaccinated group. Of these responses, 53 received more than one code, 10 received three codes, and 1 item received 4 codes.

Reflexivity Statements:

All three coders and the trainer who oversaw coding are employed by a large healthcare system in health services research. All four identify as generally pro ‘public health’ and pro ‘vaccines’ and have been required by their employer to be vaccinated against COVID-19. Additional characteristics which undoubtedly have shaped their coding are presented here. BdG is a non-Hispanic white cisgender female with a B.S. in Biochemistry, M.S. in Science and Technology Journalism and a J.D. in Law. She is a practicing Roman Catholic and although pro-vaccine states she probably wouldn’t get the flu shot annually if it wasn’t required for work unless she had another risk factor (e.g. older age). KS is a Hispanic cisgender female whose education includes a B.A. in psychology, MSW (Social Work), and Ph.D. in Social Work with experience in both academic and medical settings. She does not identify as religious and is married to a public health physician/ former Commissioner of Health. MB is a Black cisgender female with a B.S. in mathematics and a M.S. /Ph.D. in Statistics. She is a practicing non-denominational Christian. Finally, MD is a non-Hispanic White cisgender female with a B.S./M.S. in Psychology and Ph.D. in Clinical Health Psychology. She does not identify as religious and is a licensed psychologist.
